# Effect of carboxylic group on the compatibility with retarder and the retarding side effect of the fluid loss control additive used in oil well cement

**DOI:** 10.1098/rsos.180490

**Published:** 2018-09-12

**Authors:** Shenglai Guo, Yao Lu, Yuhuan Bu, Benlin Li

**Affiliations:** 1 School of Petroleum Engineering, China University of Petroleum, Qingdao, People's Republic of China; 2 Engineering Company, Xinjiang Oilfield Company, Xinjiang, People's Republic of China

**Keywords:** fluid loss control additive, cement, retarder, carboxylic group

## Abstract

The retarding side effect and the compatibility with other additives are the main problems that limit the field application of the synthesized fluid loss control additive (FLCA). The effect of the type and content of carboxylic acid groups on the retarding side effect of FLCA and the compatibility between FLCA and the retarder AMPS-IA synthesized using 2-acrylamido-2-methyl propane sulfonic acid (AMPS) and itaconic acid (IA) was studied in this paper. The type and content of carboxylic acid group have a great influence on the fluid loss control ability, the compatibility with retarder and the retarding side effect of FLCA. FLCA containing IA or maleic acid (MA) shows better compatibility with retarder than FLCA containing acrylic acid, but the retarding side effect of FLCA containing MA is weaker than that of FLCA containing IA. Thus, MA is the most suitable monomer for synthesizing FLCA having good compatibility with retarder AMPS-IA.

## 1. Introduction

Well cementing is needed after drilling of every oil and gas well has been completed; good cementing quality is the key to ensure the safe and efficient production of a well in the long term [1–4]. Normally, well cementing requires placement of the casing pipe into the well to a specified depth, and then pumping cement slurry into the annular space between the casing and formation through the casing. During well workover, filtration will occur when cement slurry is placed in contact with a permeable formation [5–7]. During application of the cement slurry, the aqueous phase of the slurry escapes into the formation, leaving the cement particles behind. If the fluid loss of the cement slurry cannot be controlled reasonably, the viscosity of the cement slurry will increase excessively, leading to an increased risk of failure of the cementing operation. In addition, the invasion of the cement slurry filtrate into the formation will cause damage to the formation and reduced production [8].

In order to control the fluid loss of cement slurry, FLCA was introduced to cement slurry. FLCA mainly contains granular materials and various types of water-soluble polymers [8]. The polymer FLCA synthesized using an acrylamide derivative shows excellent performance and has been the most widely used [9–11]. To accomplish the cementing work and improve cementing quality, many kinds of cement additive (such as dispersant, retarder and suspending agent) have been introduced in the cement slurry [12–14]. When used in combination in the cement, the problem of compatibility is very important. However, many of the cement additives are incompatible. For example, most of the synthesized polymer FLCAs are incompatible with the retarder AMPS-IA, synthesized using the monomer 2-acrylamido-2-methyl propane sulfonic acid (AMPS) and itaconic acid (IA), when they are used together [15]. Among all types of cement retarder, the retarder AMPS-IA shows excellent high-temperature resistance, good adaptability to large temperature differences and little influence on the strength of the set cement, and thus is a retarder with excellent comprehensive performance [16–17]. However, the incompatibility between it and most FLCAs has limited its application.

The incompatibility problem between FLCA and other oil well cement additives was first reported in 2006 [18]. The fluid loss control ability of FLCA was impaired when the dispersant was introduced in the cement slurry. To improve the compatibility between FLCA and dispersant, the effect of the functional group type on the compatibility of FLCA was studied [19]. According to that study, it was concluded that the compatibility between FLCA and dispersant is mainly affected by their competitive adsorption on the surface of cement particles. After the introduction of the carboxylic acid group in FLCA, the adsorbed amount of FLCA increased when both the FLCA and the dispersant were present in the cement slurry. Therefore, introduction of a carboxylic acid group in FLCA could improve the compatibility between FLCA and dispersant. However, the effect of the type and content of the carboxylic acid group on the compatibility was not studied systematically. In addition, the carboxylic acid group was the key group of the synthetic retarder, and the type and content of the carboxylic acid group may have a great influence on the retarding side effect of FLCA. The retarding side effect of FLCA should be controlled to a very low level as far as possible, which will be convenient for the formula adjustment of the cement slurry in the field application.

Therefore, in order to obtain FLCA showing good compatibility with retarder AMPS-IA and a minimum retarding side effect, it is necessary to understand the effect of the carboxylic acid group on the performance of FLCA. In this paper, the effects of carboxylic acid monomers acrylic acid (AA), maleic acid (MA) and itaconic acid (IA) on the compatibility between FLCA and retarder were studied. In order to ensure the retarding side effect of FLCA is as low as possible, the influence of the carboxylic acid group on the retarding side effect of FLCA was also studied. The results of this research could be very significant for understanding the influence of carboxylic acid groups on the performance of FLCA, to guide the research and development of FLCA and to ensure the correct field application of FLCA.

## 2. Experimental

### 2.1. Materials

AMPS with effective weight over 98%, *N,N*-dimethylacrylamide (NNDMA) with effective weight over 98%, acrylamide (AM) with effective weight over 98%, IA with effective weight over 99%, MA with effective weight over 99.5%, AA with effective weight over 98%, sodium hydroxide (NaOH) with effective weight over 96%, ammonium persulfate (APS) with effective weight over 98%, sodium bisulfite (SOB) with effective weight over 99% and potassium persulfate with effective weight over 99.5% were obtained from Sinopharm Chemical Reagent Co. (Shanghai, China). API class ‘G’ cement was obtained from Sichuan Jiahua Enterprise (Leshan, China), and the phase composition and physical properties of class ‘G’ cement are presented in table 1.

**Table 1.** Phase composition and physical properties of class G oil well cement.

C_3_S (wt%)	C_2_S (wt%)	C_3_AC (wt%)	C_3_AF (wt%)	specific density (kg l^−1^)	specific surface area (m^2^ kg^−1^)
53.7	30.46	2.8	8.0	3.17	332

### 2.2. Method

#### 2.2.1. Synthesis of FLCA

**Figure 1.**
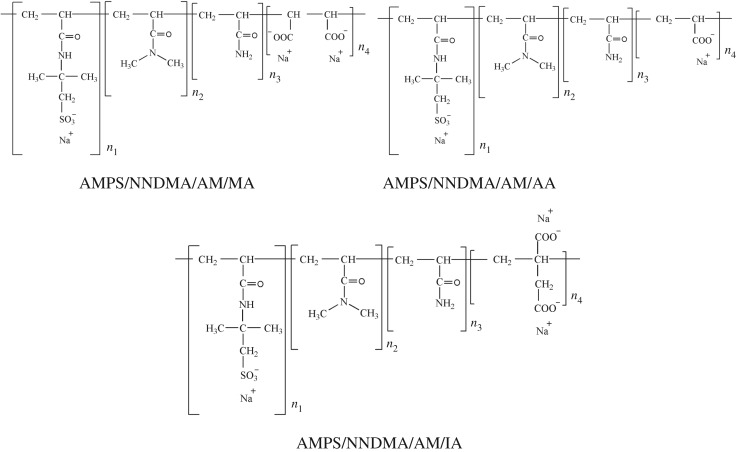
Chemical structures of synthesized FLCA.

The copolymers AMPS/NNDMA/AM/MA, AMPS/NNDMA/AM/AA and AMPS/NNDMA/AM/IA were obtained in the laboratory by the aqueous solution polymerization technique. In all of the copolymers, the feed mole ratio of AMPS/NNDMA/AM was fixed at 8/5/5, and the type and the dosage of the carboxylic acid monomers were different. The monomers were added into the solution ordinally, and the monomer concentration was 20%. The pH value was adjusted to 2 by adding NaOH solution. After the solution had been prepared, it was placed in a three-necked flask. Nitrogen gas was bubbled through the solution for 30 min with slow stirring. After the solution was heated to 60°C, the initiator (APS and SOB mass ratio 1 : 1; 0.4% (by weight of monomers)) was added to the solution drop-wise. The reaction was allowed to proceed for about 5 h. Finally, the reaction product was converted to a powder by drying and crushing. The chemical structures of the FLCAs are illustrated in figure 1.

#### 2.2.2. Synthesis of retarder AMPS-IA

**Figure 2.**
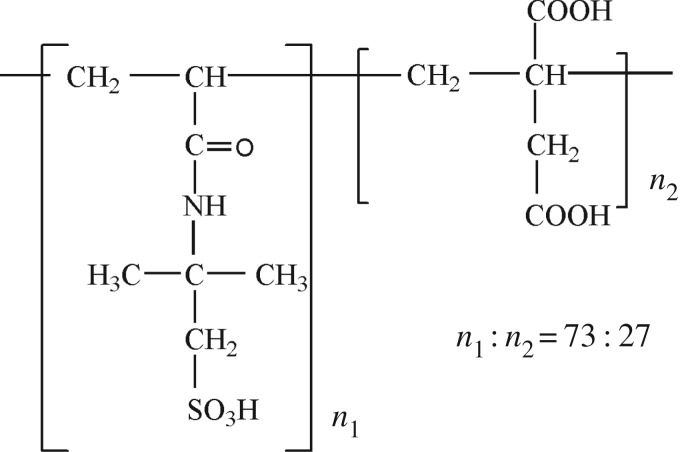
Chemical structure of synthesized retarder AMPS-IA.

The copolymer AMPS-IA was synthesized by aqueous free radical copolymerization. The feed molar ratio of AMPS and IA was 73 : 27. The monomers were added into the solution ordinally, and the monomer concentration was 20%. The pH value was 1–2 naturally generated by the dissolution of the monomers. After the solution had been prepared, it was placed in a three-necked flask. Nitrogen gas was bubbled through the solution for 30 min with slow stirring. After the solution was heated to 60°C, the initiator (potassium persulfate; 1% (by weight of monomers)) was added drop-wise to the solution. The reaction was allowed to proceed for about 5 h. Finally, the reaction product was converted to a powder by drying and crushing. The chemical structure of retarder AMPS-IA is illustrated in figure 2.

#### 2.2.3. Cement slurry preparation

Cement slurries were prepared in accordance with the test procedure set out in API Recommended practice for testing well cements 10B [20]. The mixing device for preparation of slurries should be a one-quart sized, bottom-drive, blade-type mixer. The dry materials should be weighed and uniformly blended prior to being added to the mixing fluid. The mix water and any liquid additives should be mixed uniformly and placed on the mixer base. The dry materials should be added in the mixer within 15 s at a uniform rate of 4000 ± 200 r.p.m. After that, the rotation speed should be increased to 12 000 ± 500 r.p.m. for 35 s. To prepare the cement slurry, FLCA (1% by weight of cement (BWOC)), retarder (0 or 0.5% BWOC) and anti-foaming agent (0.5% BWOC) were added into the class ‘G’ cement. The cement slurry was prepared with a water-to-cement ratio (W/C) of 0.44.

#### 2.2.4. API static fluid loss

According to the standard ‘Recommended practice for testing well cements' [20], a stirred fluid loss cell was used to obtain the API fluid loss. After the cement slurry had been prepared, it was poured into the stirred fluid loss cell. While agitating with the paddle, the slurry was heated according to the scheduled requirement. The slurry was stirred continually at a speed of 150 ± 15 r.p.m. before it reached the final temperature. Filtration was obtained by passing through a 325 mesh metal sieve (3.5 in. (88.9 mm)). 1000 psi (6.9 MPa) differential pressure was applied to the fluid loss cell. The filtrate produced by the differential pressure was collected for 30 min. The fluid loss was twice as much as the collected filtrate volume.

#### 2.2.5. Initial setting time of the cement slurry

The measurement of setting time was performed using Vicat apparatus according to the European norm [21]. The Vicat apparatus consists of a frame bearing a movable plunger of 300 g weight. The plunger is 10 mm in diameter at the upper end and is fitted with a removable 1 mm diameter needle at the bottom end. The plunger is reversible and carries an indicator which moves over the scale graduated in millimetres. The method is based on measuring the depth of penetration of a steel right cylinder needle into the cement paste. The time between starting time and the time at which the distance between the needle and the base-plate of the slurry is *d* = 6 ± 3 mm, measured to the nearest minute, is defined as the initial setting time (IST) of the cement.

#### 2.2.6. Intrinsic viscosity number

An Ubbelohde viscometer was used to measure intrinsic viscosity number (*η*) of FLCA with 1 mol l^−1^ sodium chloride solution at 30°C. The concentration of the FLCA was 0.0005 g ml^−1^. After the flow time was tested, the ‘one point method’ was used to calculate the intrinsic viscosity number (*η*). To make the result more accurate, an average was obtained after the test was repeated three times.

## 3. Results and discussion

### 3.1. Effect of carboxylic group on the fluid loss control ability of FLCA

**Figure 3.**
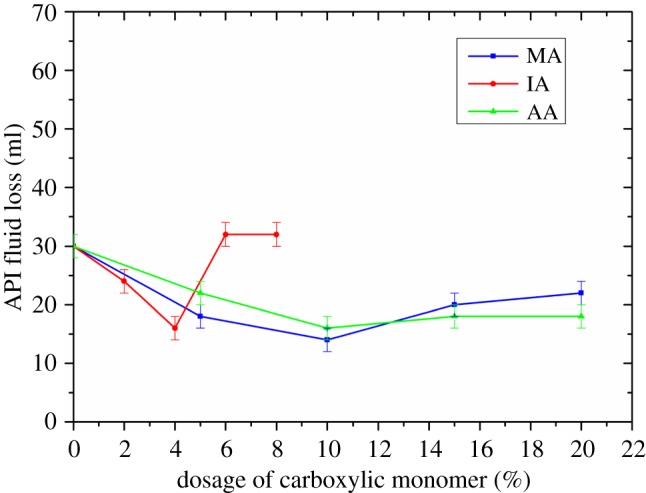
The effect of carboxylic group on the fluid loss control ability of FLCA.

Based on the molar ratio of AMPS/NNDMA/AM being fixed at 8/5/5, different FLCAs were synthesized using MA, IA or AA with the method described in §2.2.1. API fluid loss of the cement slurry containing the FLCA but without retarder AMPS-IA was tested using the method mentioned in §2.2.4. Figure 3 shows the effect of carboxylic group on the fluid loss control ability of FLCA.

In figure 3, the API fluid loss of the cement slurry without retarder first decreased and then increased with the increase of the mole ratio of carboxylic group. The minimum fluid loss of cement slurry was 14 ml, 16 ml and 16 ml when the carboxylic group was MA, IA and AA, respectively, and thus the type of carboxylic group has less influence on the fluid loss control ability of FLCA.

**Figure 4.**
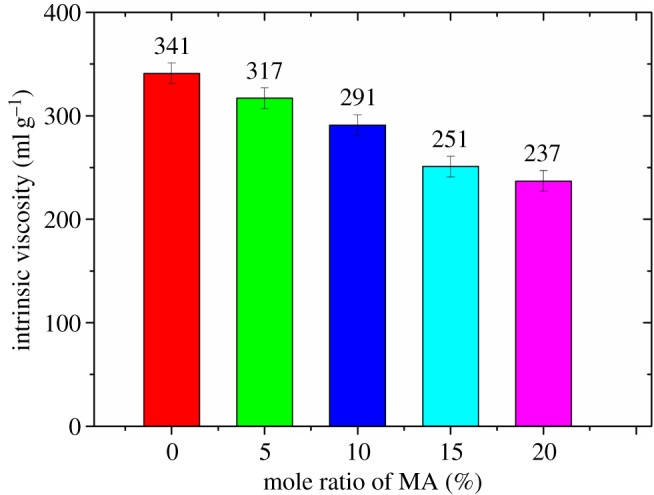
The effect of the mole ratio of MA on the intrinsic viscosity of FLCA.

**Figure 5.**
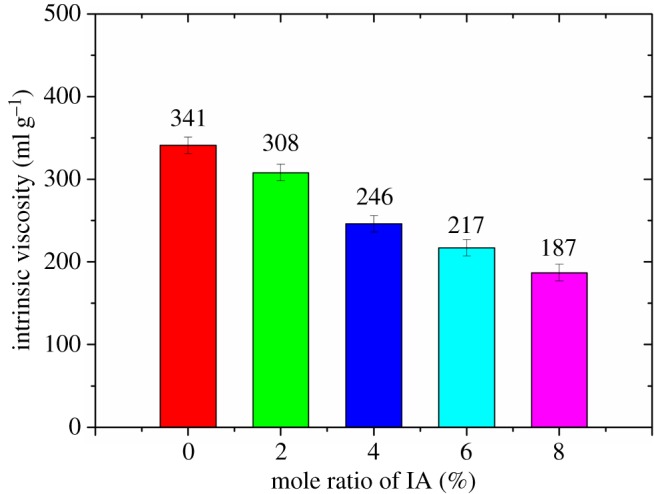
The effect of the mole ratio of IA on the intrinsic viscosity of FLCA.

**Figure 6.**
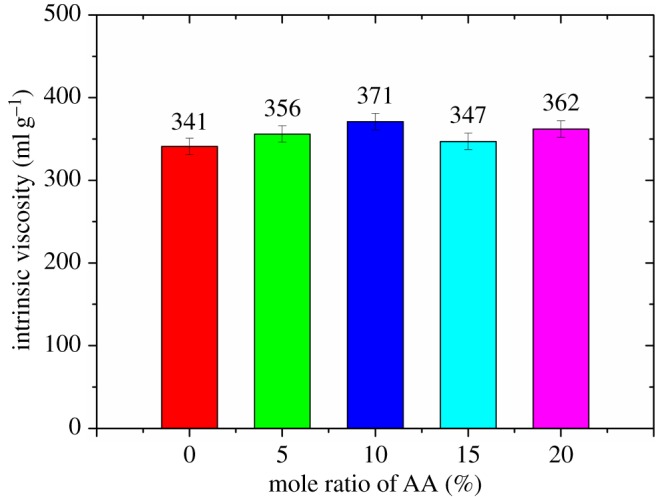
The effect of the mole ratio of AA on the intrinsic viscosity of FLCA.

At first, the fluid loss of cement slurry without retarder gradually decreases with the increase of the mole ratio of carboxylic group, which could be attributed to the adsorption capacity of the FLCA gradually being enhanced with the increase of the mole fraction of carboxylic group [18]. However, after the mole ratio of carboxylic group surpasses some value, an increase in the fluid loss of the cement slurry is mainly related to the decrease in the molecular weight of FLCA (the molecular weight could be expressed by the intrinsic viscosity as shown in figures 4–6). Owing to the intrinsic viscosity of FLCA containing AA changing little after the dosage of AA surpasses 10%, the API fluid loss shows little change.

### 3.2. Effect of carboxylic group on the compatibility between retarder AMPS-IA and FLCA

**Figure 7.**
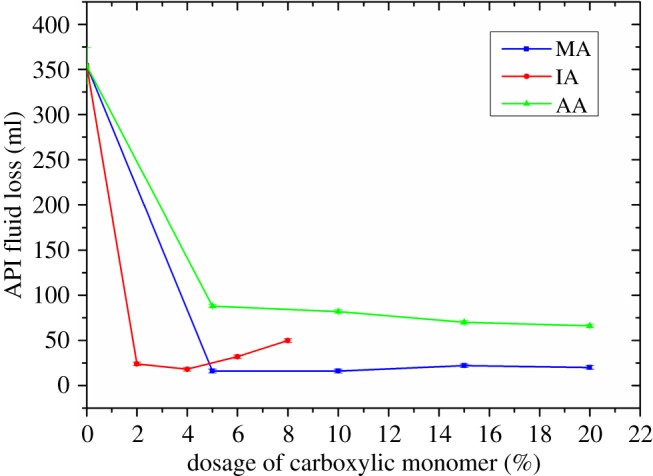
The effect of carboxylic group on the compatibility between retarder AMPS-IA and FLCA.

The effect of the carboxylic group on the compatibility of FLCA with retarder AMPS-IA was studied by testing the API fluid loss of the cement slurry containing both 1% FLCA and 0.5% retarder AMPS-IA. Figure 7 shows the effect of carboxylic group on the API fluid loss of the cement slurry.

In figure 7, the API fluid loss was very high when no carboxylic group was introduced in FLCA. After the introduction of the carboxylic group, the API fluid loss decreased noticeably, which shows the significant effect of the carboxylic group on the compatibility between retarder AMPS-IA and FLCA. Although the API fluid loss decreases noticeably after the introduction of each of MA, IA and AA, the fluid loss control ability of FLCA containing MA, IA or AA is different. The cement slurry containing FLCA with MA shows the minimum API fluid loss and the API fluid loss changes little with the dosage increase of MA, which shows the optimal compatibility with retarder AMPS-IA. The cement slurry containing FLCA with IA also shows preferable fluid loss control ability when the dosage of IA is between 2% and 4%; however, the API fluid loss increases obviously after the IA dosage surpasses 4%. Although API fluid loss of the cement slurry containing FLCA with AA also decreases obviously after the introduction of AA, the API fluid loss is still very high compared with FLCA containing MA or IA.

After the introduction of a carboxylic group, the improvement of compatibility between retarder AMPS-IA and FLCA could be attributed to the adsorption capacity of the FLCA being enhanced with the introduction of the carboxylic group [18]. Owing to the surface affinity of –vic–(COO^–^)_2_ which existed in MA and IA being stronger than –COO^–^ which existed in AA, the fluid loss control ability of FLCA containing AA is weaker than that of FLCA containing IA or MA in the cement slurry containing 0.5% retarder AMPS-IA [19]. After the dosage of carboxylic group surpassed 4%, the increase in the API fluid loss of the cement slurry containing FLCA with IA may be attributed to the molecular weight (as shown in figure 5) of the FLCA being too low to control the fluid loss well.

### 3.3. Effect of carboxylic group on the retarding side effect of FLCA

**Figure 8.**
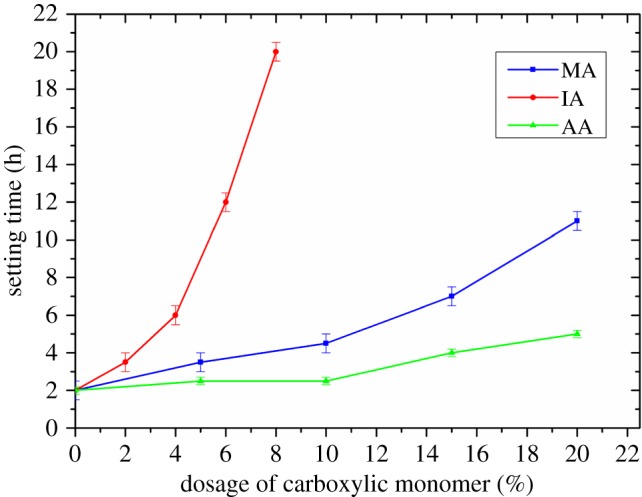
The effect of carboxylic group on the retarding side effect of FLCA.

The effect of the carboxylic group on the retarding side effect of FLCA was studied by testing the setting time of the cement slurry containing 1% FLCA but without retarder AMPS-IA. Figure 8 shows the effect of carboxylic group on the setting time of the cement slurry.

In figure 8, the setting time of the cement slurry increases with the increase of the mole ratio of carboxylic group, but the degree of increase is different. The setting time of the cement slurry containing FLCA with IA increases sharply, which is very adverse to the formula adjustment of the cement slurry in the field application. The setting time of the cement slurry containing FLCA with MA or AA increases gently, which is beneficial for adjusting the cement slurry formula.

In general, the FLCA containing MA shows better fluid loss control ability, the best compatibility with retarder AMPS-IA, and a moderate retarding side effect, and MA is the best carboxylic monomer for synthesizing an FLCA with excellent performance.

### 3.4. Effect of temperature on the fluid loss control ability of FLCA

**Figure 9.**
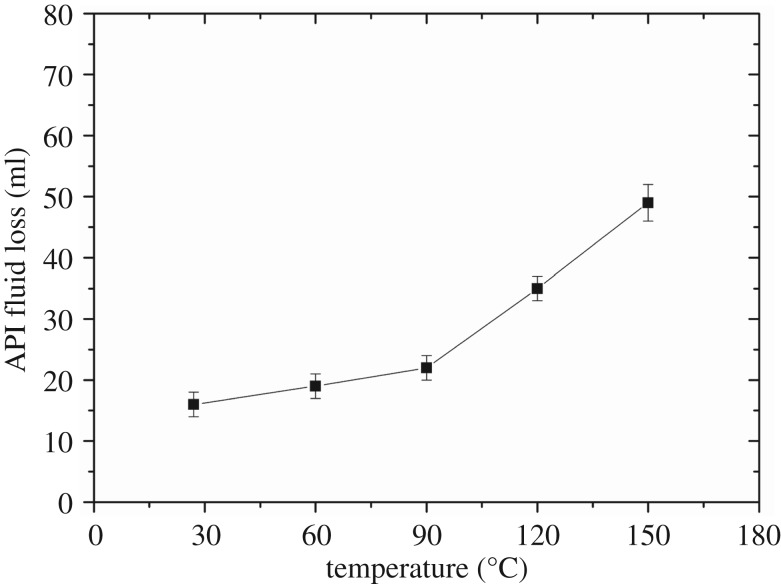
The effect of temperature on the fluid loss control ability of FLCA.

The temperature of the wellbore increases as the depth of the well increases. Normally, the temperature of the wellbore can reach around 150°C. The fluid loss of the slurry under high temperatures is very important to the cementing operation security, and the effect of temperature on the fluid loss of slurry is obvious. Therefore, the effect of temperature on the fluid loss control ability of the FLCA was evaluated. MA was chosen as the carboxylic monomer to synthesize the FLCA. The feed mole ratio of AMPS/NNDMA/AM/MA was 4/2.5/2.5/1. The effect of temperature on the fluid loss control ability of FLCA was studied by testing the API fluid loss of the cement slurry containing both 1% FLCA and 0.5% retarder AMPS-IA. Figure 9 shows the API fluid loss under different temperatures.

The API fluid loss of the cement slurry increased as the temperature increased (as shown in figure 9). However, even if the temperature attained 150°C, the API fluid loss could be still controlled below 50 ml, which is very beneficial to the cementing operation security and the prevention of gas channelling. The increase of the API fluid loss as the temperature increases could be attributed to the decrease in the amount of adsorption of the FLCA on the cement particles as the temperature increases [22].

## 4. Conclusion

(1) The type and content of the carboxylic acid group have a great influence on the fluid loss control ability, the compatibility with retarder and the retarding side effect of FLCA.
(2) FLCA containing IA or MA shows better compatibility with retarder than FLCA containing AA, but the retarding side effect of FLCA containing MA is weaker than that of FLCA containing IA, so MA is the most suitable monomer for synthesizing FLCA having good compatibility with retarder AMPS-IA.
(3) The compatibility between retarder AMPS-IA and FLCA could be attributed to the adsorption capacity of the FLCA being enhanced with the introduction of the carboxylic group. Owing to the surface affinity of –vic–(COO^–^)_2_, which existed in MA and IA, being stronger than –COO^–^ which existed in AA, the fluid loss control ability of FLCA containing AA is weaker than that of FLCA containing IA or MA when the cement slurry contains 0.5% retarder AMPS-IA.
(4) The molecular weight of FLCA also shows some influence on the compatibility between FLCA and retarder AMPS-IA.
(5) The FLCA containing MA also shows excellent fluid loss control ability under high temperature.

## Data accessibility

Our data are deposited at the Dryad Digital Repository: http://dx.doi.org/10.5061/dryad.42v01vc [23].
